# Triterpenoid resinous metabolites from the genus *Boswellia*: pharmacological activities and potential species-identifying properties

**DOI:** 10.1186/1752-153X-7-153

**Published:** 2013-09-12

**Authors:** Yuxin Zhang, Zhangchi Ning, Cheng Lu, Siyu Zhao, Jianfen Wang, Baoqin Liu, Xuegong Xu, Yuanyan Liu

**Affiliations:** 1 School of Chinese Materia Medica, Beijing University of Chinese Medicine, Beijing, China; 2 Institute of Basic Research in Clinical Medicine, China Academy of Chinese Medical Sciences, Beijing, China; 3 Zhengzhou Hospital of Traditional Chinese Medicine, Zhengzhou, China

**Keywords:** *Boswellia*, Triterpenoids, Anti-inflammation, Anti-carcinogenic, Pharmacological activities, Bioavailability

## Abstract

The resinous metabolites commonly known as frankincense or olibanum are produced by trees of the genus *Boswellia* and have attracted increasing popularity in Western countries in the last decade for their various pharmacological activities. This review described the pharmacological specific details mainly on anti-inflammatory, anti-carcinogenic, anti-bacterial and apoptosis-regulating activities of individual triterpenoid together with the relevant mechanism. In addition, species-characterizing triterpenic markers with the methods for their detection, bioavailability, safety and other significant properties were reviewed for further research.

## Introduction

The resins commonly known as frankincense or olibanum, which are obtained from trees of the genus *Boswellia*, are the best-known of the aromatic gum resins used throughout the world as incense in religious ceremonies [1]. Moreover, frankincense has enormous socio-economic importance. Historically, it has been used as incense in religious and cultural ceremonies and as an ingredient in traditional medicines since time immemorial, whereas currently, it is widely used as an adhesives agent, as an ingredient in cosmetic preparations, as a fragrance used in daily rituals, and as a coating for materials. The medicinal properties of frankincense have also been studied to shed light on its potential use in treating or curing diseases [2,3]. The medicinal use of frankincense may be among mankind’s oldest therapies [4], and in the contemporary era, it is still used extensively in regions ranging from North Africa to China. Frankincense has been demonstrated to have curative activity when used within the system of traditional medicine called Ayurveda, which is practiced in India; however, it has fallen into obscurity during the present era of synthetic drugs [5].

Much attention has been focused on the beneficial effects of frankincense as a medicinal component of therapies targeting allergic asthma, inflammatory bowel diseases, rheumatoid arthritis and osteoarthritis, brain tumors and edema [6]. Meanwhile, it is also used in the treatment of amenorrhea, menorrhagia, polyuria, scrofula, syphilis, asthma, bronchitis, sores, and diseases of the nervous system [7]. Generally speaking, frankincense is widely used as an anti-inflammatory drug and numerous reports suggest that triterpenoids may be the bioactive ingredients responsible for these properties [6,8]. In addition, pure compounds isolated from frankincense have anti-neoplastic activity. Many studies have demonstrated the potent anti-neoplastic activities of triterpenoids acids obtained from frankincense, and especially boswellic acids (BAs) and their derivatives. These compounds have been shown to possess potential chemoprevention effects in prostate cancer [8,9], cervical cancer [10], breast cancer [11], colorectal cancer [12], pancreatic cancer [13] and bladder cancer [14]. In light of what is known of the medicinal properties of these metabolites, the present review places special emphasis on the pharmacological activities of the characteristic components of frankincense, such as acetyl- 11-keto-β-boswellic acid (AK-β-BA) (4). Aside from the natural resinous metabolites, we also review resin-derived compounds that have been shown to possess conspicuous pharmacological activities following modification of their structures (48–53) as these may provide new leads for future pharmaceutical research aimed at developing and synthesizing novel analogs with desired characteristics.

Given the diverse pharmacological activities of the compounds present in frankincense, it is important to characterize the pharmacological activities of frankincense harvested from different species and to identify species-specific differences in activity and therapeutic potential. On the basis of published studies, we review the triterpenoid resinous metabolites that are present in frankincense obtained from several different *Boswellia* species with respect to their medicinal properties. In our review of triterpenoid activities, structures and species-specific profiles, we have attempted to include all of the triterpenoids from frankincense that have been studied to date. To draw distinctions between the resins from various species of *Boswellia* in terms of their medicinal potential, the pharmacological activities and the primary mechanisms of action of the characteristic triterpenoids are discussed as far as possible.

## Identification of *Boswellia* species

### Various sources

Frankincense exudes from incisions in the bark of trees of the *Boswellia* (*Burseraceae*) genus and approximately 25 species belonging to this genus are used sources of this resin. These species are widely distributed in India, Arabia and the northeastern coast of Africa. As has been reported previously, the trees referred to as “true frankincense-producing trees” may in fact be any of several main *Boswellia* species depending on the geographical location in question [4,15-17], and these species are listed below.

♦ *Boswellia sacra* Flueck. (found in Southern Arabia) is known in Arabia as “maghrayt d’sheehaz”, and the resin it produces is known as “lubãn dhakar”.

♦ *Boswellia carterii* Birdw. (found in Somalia and Southern Arabia) is better known as “Mohr”. This specie is also found in Sudan and, in rare cases, in Yemen. *Boswellia bhau-dajiana* Birdw. (found in Somalia), whose local name is “Mohr-add”, has recently been found to be identical to *B. carterii*. Generally, the resins that both species produce are called “lobãn dakar” or are more commonly referred to as “beeyo” quality.

♦ *Boswellia frereana* Birdw. (found in Somalia) produces the most expensive type of olibanum on the market, and this resin is known as “lobãn majdi” or, more commonly, as “maydi”.

♦ *Boswellia papyrifera* Hochst. (found in East Africa), another deciduous, gum-producing, multipurpose perennial tree that grows in Somalia, Ethiopia, Eritrea, Sudan and in the other east African countries, is claimed to have been the source of olibanum in antiquity and produces resin of a quality known as “boido” [4,7,18].

♦ *Boswellia serrata* Roxb. (found in East India) is also known as “Indian olibanum” and is found in the central and northern parts of east India [3]. This specie produces olibanum resins of various qualities, which are commonly known as “salai guggul”.

In spite of the diversity in the species from which frankincense is obtained and their broad and often overlapping geographical ranges, the gum-producing trees are generally referred to as follows: B. papyrifera (“African frankincense”), B. frereana (“African frankincense”), B. serrata (“Indian Frankincense”) and B. carterii/B. sacra (“Arabian frankincense”) [19-21]. However, it should be noted that there are divergent opinions concerning *B. carterii* that have led to some researchers referring to this specie as “African frankincense” [21].

The quality and the commercial value of resins differ based on the species from which they are obtained. In general, the finest and most expensive frankincense is maydi from *B. frereana*, while the most common is beeyo from *B. carterii or Boswellia bhau-dajiana* Birdw [22]. Thus, the identification of species-specific marker profiles would be a significant advance that could provide a rapid way to distinguish the different types and sources of frankincense, and this could be used in a commercial context to appraise resins.

### The characteristic triterpenic compounds of *Boswellia* species

As detailed above, the triterpenic compounds have been shown to have potential as markers that can identify the source of frankincense. Below is a summary of the characteristic triterpenic compounds of various *Boswellia* species.

♦ *B. carterii* and *B. sacra* olibanum have quite similar chemical compositions and are characterized by the presence of lupeolic acid (25), BAs and their respective O-acetyl derivatives [23,24].

♦ *B. serrata* is characterized by the presence of tirucallane [25] and euphane [26] skeletons, which are not commonly found in *Boswellia* species, in addition to BAs and their respective O-acetyl derivatives.

♦ *B*. *frereana* is characterized by the presence of lupeol (29) and 3-epi-lupeol (28) [23,27] in conjunction with triterpenes with dammarane [28] skeletons. Lupeolic acid (25), BAs (1, 15) and their respective O-derivatives (26, 2 and 16) are not found in frankincense from these species [23].

**Figure 1:**
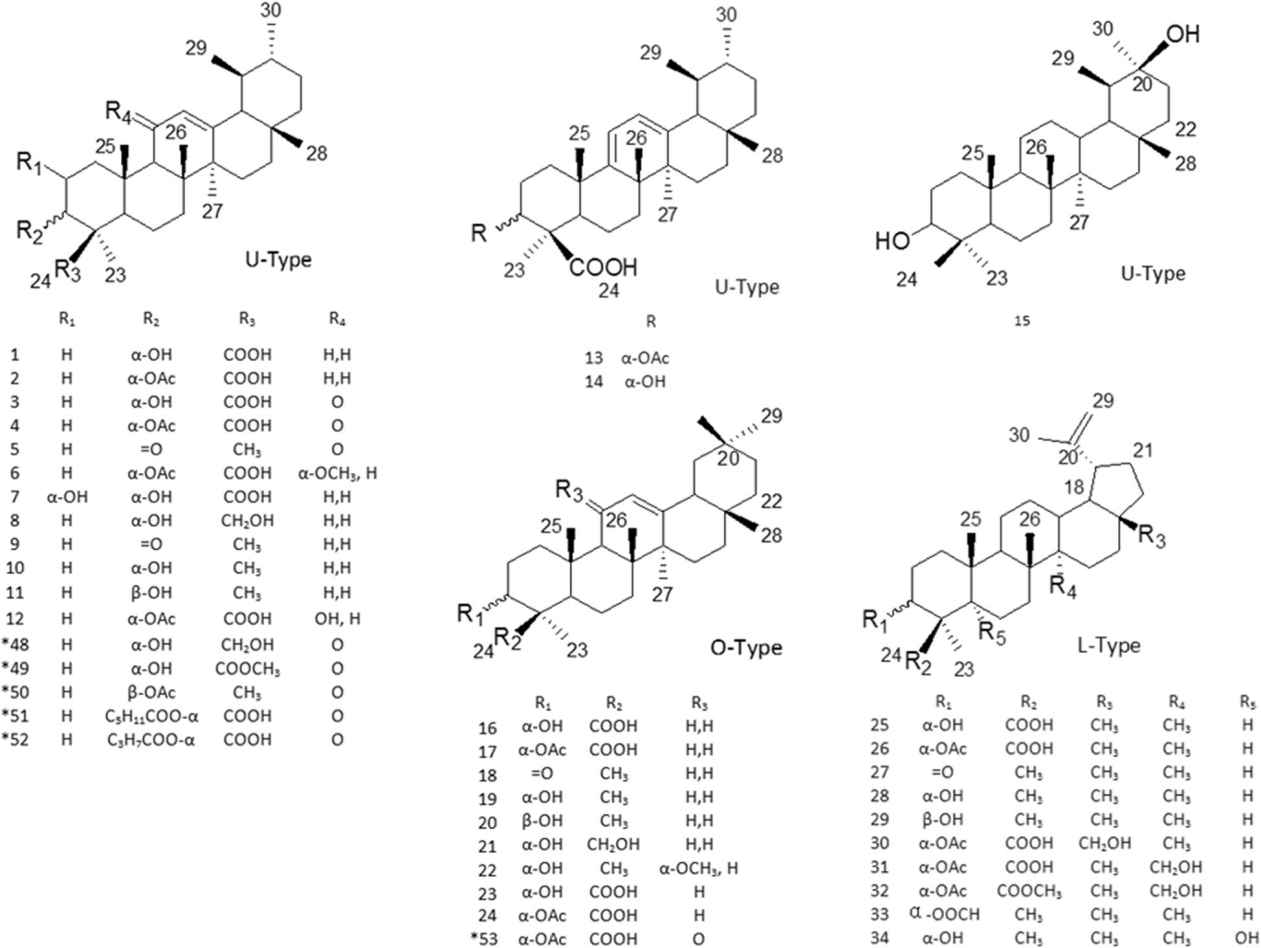
Structures of triterpenoids 1–34, 48–53.

**Figure 2:**
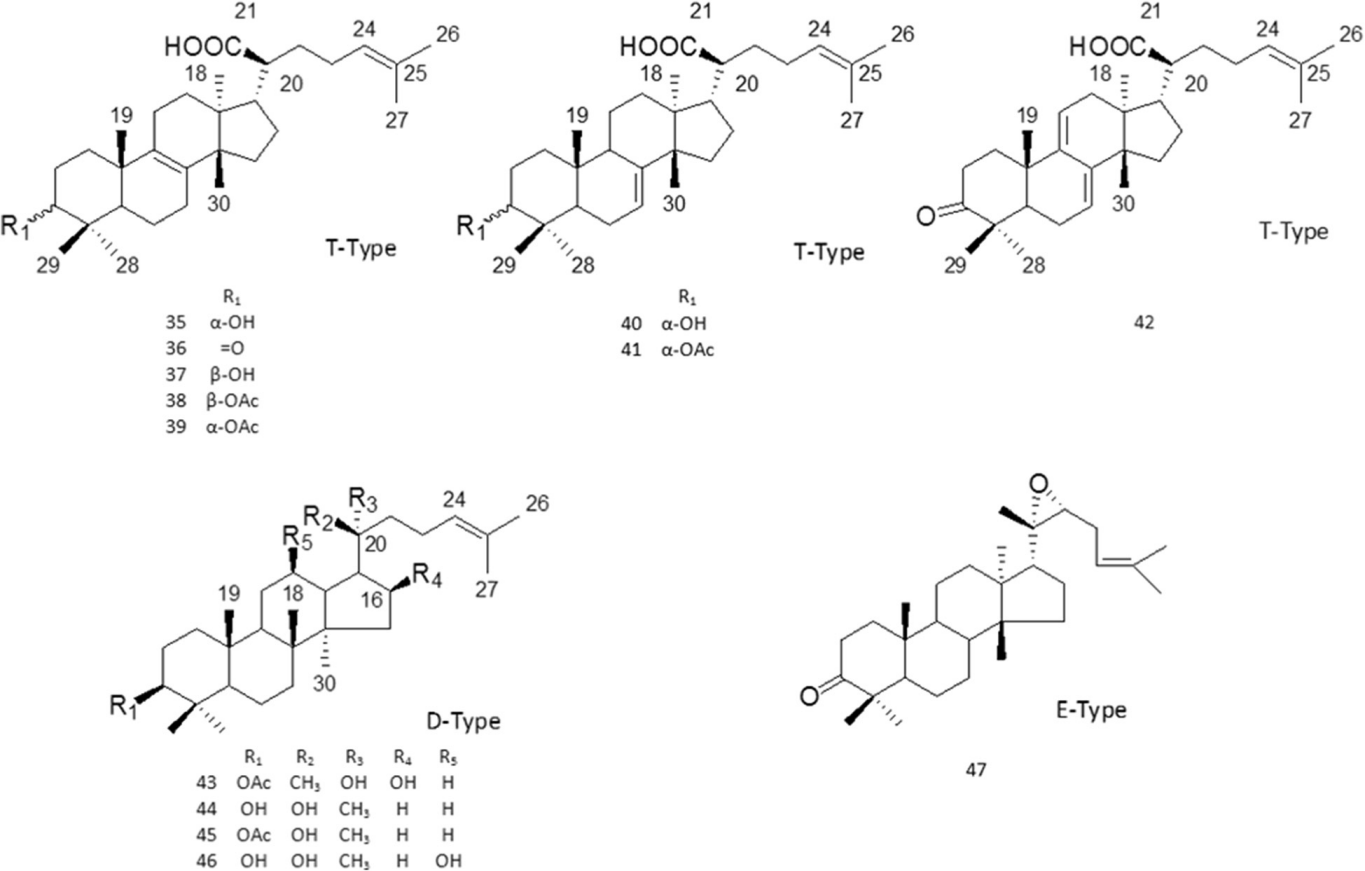
Structures of triterpenoids 35–47.

Further details of the triterpenoid content of frankincense from the various *Boswellia* species are summarized in Table 1. The natural resinous triterpenoids can be grouped into the following 6 types: ursane-type triterpenes (type U), oleanane-type triterpenes (type O), lupane-type triterpenes (type L) and tirucallane-type triterpenes (type T), dammarane-type triterpenes (type D) and euphane-type triterpenes (type E). Their corresponding structures are shown in Figure 1 and Figure 2. Type U, type O and type L belong to the pentacyclic triterpenes class, while type T, type D and type E are classified as tetracyclic triterpenes. Compounds 1–15 and 48–52 are of type U and are divided into three subclasses based on the number of double bonds attached to the aromatic nucleus. Compounds 1–12 and 48–52, all possess one double bond at C-12 and a subset of these also have a carbonyl group at C-11. Compounds 13 and 14 have two double bonds located at C-9 (11) and C-12 (13), respectively, and do not have a carbonyl group, while compounds 15 are saturated. Compounds 16–24 and 53 are of type O and the skeletons of them possess one or two double bonds attached to the aromatic nucleus. Compounds 35–42 which belong to the type T are divided into three sections in the light of the number and the positions of double bonds in *aromatic* nucleus*.* Moreover, compounds 25–34, 43–46 and 47 are classified as type L, type D and type E respectively.

**Table 1:** **Characteristic triterpenoids in frankincense harvested from various *Boswellia *species**

**Type**	**No.**	**Common name**	**Systematic name**	**Resource**	**Ref.**
**Pentacyclic triterpenes**					
U	1	β-Boswellic acid (β-BA)	3α-hydroxy-urs-12-en-24-oic acid	*B .carterii, B. sacra, B. serrata*	[18,29,30]
	2	3-acetyl-β-BA (Aβ-BA)	3α-O-acetyl-urs-12-en-24-oic acid	*B. serrate, B .carterii*	[5,29]
	3	11-keto-β-BA (Kβ-BA)	3α-hydroxy-11-oxo-urs-12-en-24-oic acid	*B. carterii, B. serrata*	[29,31]
	4	3-acetyl-11-keto-β-BA (AKβ-BA)	3α-O-acetyl-11-oxo-urs-12-en-24-oic acid	*B. carterii, B. serrata*	[31,32]
	5	12-ursene-2-diketone	urs-12-en-3,11- diketone	*B. serrata*	[33]
	6	3-acetyl-11α-methoxy-β –BA	3α-O-acetyl-11α-methoxy-urs-12-en-24-oic acid	*B. carterii*	[34]
	7	\	2α,3α-dihydroxy-urs-12-en-24-oic acid	*B. serrata.*	[35]
	8	urs-12-en-3α,24-diol	3α, 24-dihydroxyurs-12-ene	*B. serrata.*	[35,36]
	9	α-amyrenone	urs-12-en-3-one	*B. carterii, B. serrata*	[27,37]
	10	3-epi-α-amyrin	3α-urs −12-en-3-ol	*B. carterii, B. serrata*	[27]
	11	α-amyrin	3β-urs-12-en-3-ol	*B. carterii, B. serrata*	[27,38]
	12	3-acetyl-11-hydroxy-BA	3α-O-acetyl-11-hydroxy-urs-12-en-24-oic acid	*B. serrata*	[39]
	13	3-acetyl-9,11-dehydro-β-BA	3α-O-acetyl-9,11-dehydro-urs-12-en-24-oic acid	*B. carterii*	[31]
	14	9,11-dehydro-β-BA	3α-hydroxy-9,11-dehydro-urs-12-en-24-oic acid	*B. carterii*	[31]
	15	18Hα,3β,20β-ursanediol	3, 20β-dihydroxy-urs-3-ol	*B. carterii*	[40]
	*48	11-keto-diol	3α,24-dihydroxy-11-oxo-urs-12-ene	*	[41]
	*49	11-keto-β-BA methyl ester	3α-11-oxo-urs-12-en-24-oic acid methyl ester	*	[41]
	*50	acetyl-11-keto-amyrin	3β-acetyl-11-oxo-urs-12-ene	*	[41]
	*51	hexanoyloxy-11-keto-β-BA (HKBA)	3α-O-n-hexanoyl-11-oxo-urs-12-en-24-oic acid	*	[42]
	*52	butyryloxy-11-keto-β-BA (BKBA)	3α-O-n-butyryl-11-oxo-urs-12-en-24-oic acid	*	[42,43]
O	16	α-Boswellic acid (α-BA)	3α-hydroxy-olean-12-en-24-oic acid	*B. carterii, B. serrata*	[18]
	17	3-acetyl α-BA(Aα-BA)	3α-O-acetyl-olean-12-en-24-oic acid	*B. serrate, B. carterii*	[29,44]
	18	β-amyrenone	olean-12-en-3-one	*B. carterii, B. serrata*	[18]
	19	3-epi-β-amyrin	3α-olean-12-en-3-ol	*B. carterii, B. serrata*	[18]
	20	β-amyrin	3β-olean-12-en-3-ol	*B. carterii, B. serrata*	[18,45]
	21	\	3α,24-dihydroxy-olean-12-ene	*B. serrata*	[10,36]
	22	olibanumol E	3α-hydroxy-11-methoxyl-olean-12-ene	*B. carterii*	[46]
	23	9,11-dehydro-α-BA	3α-hydroxy-9,11-dehydro-olean-12-en-24-oic acid	*B. serrata*	[47]
	24	3-acetyl-9,11-dehydro-α-BA	3α-O-acetyl-9,11-dehydro-olean-12-en-24-oic acid	*B. serrata*	[44]
	*53	3-acetyl-11-keto-α-BA	3α-O-acetyl-11-oxo-olean-12-en-24-oic acid	*	[48]
L	25	lupeolic acid	3α-hydroxy-lup-20(29)-en-24-oic acid	*B. carterii*	[21]
	26	acetyl-lupeolic acid	3α-O-acetyl-lup-20(29)-en-24-oic acid	*B. carterii*	[21]
	27	lupenone	lup-20(29)-en-3-one	*B. frereana*	[18]
	28	epi- lupeol	3α-lup-20(29)-en-3-ol	*B. frereana*	[18]
	29	lupeol	3β-lup-20(29)-en-3-ol	*B. frereana, B. carterii*	[18,29]
	30	3-acetyl-28-hydroxy-lupeolic acid	3α-O-acetyl-28-hydroxy-lup-20(29)-en-24-oic acid	*B. carterii*	[49]
	31	3-acetyl-27-hydroxy-lupeolic acid	3α-O-acetyl-27-hydroxy-lup-20(29)-en-24-oic acid	*B. papyrifera*	[7]
	32	methyl-3α-O-acetyl-27-hydroxy-lupeolic acid	3α-O-acetyl-27-hydroxy-lup-20(29)-en-24-oate	*B. papyrifera*	[7]
	33	olibanumol F	3α-lup-20(29)-en-3- acid ester	*B. carterii*	[46]
	34	olibanumol G	3α,5α-dihydroxy-lup-20(29)-en-24-oic acid	*B. carterii*	[46]
**Tetracyclic triterpenes**					
T	35	α-elemolic acid	3α-hydroxy-tir-8,24-dien-21-oic acid	*B. carterii, B. serrata*	[25,50]
	36	elemonic acid (3-oxo-tirucallic acid)	3-oxo-tir-8,24-dien-21-oic acid	*B. carterii, B. serrata*	[9,25,29]
	37	β-elemolic acid	3β-hydroxy-tir-8,24-dien-21-oic acid	*B. carterii, B. serrata*	[25,29,50]
	38	3β-acetoxy-tireucallic acid	3β-O-acetyl-tir-8,24-dien-21-oic acid	*B. carterii*	[9]
	39	3α-acetoxy-tirucallic acid(B)	3α-O-acetyl-tir-8,24-dien-21-oic acid	*B. serrata*	[25,51]
	40	\	3α-hydroxy-tir-7,24-dien-21-oic acid	*B. carterii*	[50]
	41	3α-acetoxy-tirucallic acid(A)	3α-O-acetyl-tir-7,24-dien-21-oic acid	*B. carterii*	[9,50]
	42	\	3-oxo-tir-7,9(11),24-trien-21-oic acid	*B. carterii*	[40]
D	43	\	3β-O-acetyl-16(S),20(R)-dihydroxy-dammar-24-ene	*B. frereana*	[28,52]
	44	\	3β,20(S)-dihydroxy-dammar-24-ene	*B. frereana*	[28]
	45	\	3β-O-acetyl-20(S)-hydroxy-dammar-24-ene	*B. frereana*	[28]
	46	20(S)-protopanaxadiol	3β-hydroxy-12β,20(S)-dihydroxy-dammar-24-ene	*B. frereana*	[28]
E	47	\	20,22-epoxyeupha-24-ene-3-one	*B. serrata*	[26]

* Artificially synthesis.

### Species-characterizing triterpenic markers and methods

The ability to identify the source of frankincense may lead to improvements in the quality of the products produced using this resin. A rapid means of identifying the source of frankincense on the basis of characteristic markers of each species is necessary to achieve this end. Such an identification method will also be very useful in efforts to characterize the presence of resins from different species in archaeological samples. In early studies, attention was focused on the diterpenes and volatiles, which have been regarded as the primary diagnostic markers in generic assays, and the methods used consisted of GC, GC-MS, pyrolysis-GC-MS, solid phase micro extraction and thin layer chromatography (TLC). However, other published studies provide evidence of the utility of the triterpenoid resinous metabolites as species-specific markers [22,23]. The primary methods used to detect these markers are TLC and high performance liquid chromatography (HPLC).

### Differentiating *B. frereana*-produced resins from *B. carterii* and *B. sacra*-produced resins

A reversed-phase high performance liquid chromatographic procedure (RP-HPLC) has been developed that is aimed at differentiating between resins obtained from different species [23]. Testing of this method using resins with known botanical origins [17] demonstrated that it was not able to reliably distinguish *B. carterii* resin from that of *B. sacra* because the resins of these two species have similar chemical compositions that result in qualitatively similar chromatograms. Nevertheless, this chromatographic method is able to distinguish the finest and most expensive Somalian olibanum resin produced by *B. frereana* from the other common Arabian and African resins produced by *B. sacra* and *B. carterii*. In fact, *B. frereana* resin produced a characteristic chromatographic profile in which 3-epi-lupeol (28) was the most prominent peak, while lupeolic acid (25), boswellic acids (1, 15) and their respective O-derivatives (26, 2 and 16) were not detected [23]. The absence of these latter compounds is surprising because they have been found to be the major components in frankincense resins analyzed previously. In summary, *B. carterii* and *B. sacra* resins are characterized by the presence of lupeolic acid (25), BAs and their respective O-acetyl derivatives, whereas 3-epi-lupeol (28) in methanolic extracts can be considered to be a marker that is characteristic of *B. frereana* resins. Thus, these findings form the basis of a simple method for differentiating resins produced by *B. carterii* or *B. sacra* from those produced by *B. frereana*. Moreover, this method could be used in a commercial context to distinguish common olibanum (beeyo) from the finest and the most expensive frankincense (maydi) [23].

### Differentiating *B. papyrifera-*produced resins from *B. carterii* and *B. sacra*-produced resins

A normal phase TLC method has been developed which is able to distinguish between resins produced by *B. carterii* or *B. sacra* and those obtained from *B. papyrifera*. An eluent consisting of a mixture of pentane and diethyl ether (2:1) with 1% acetic acid is used in conjunction with anisaldehyde dyeing reagent in this method, and the resulting chromatograms are analyzed by UV detection (254 nm) [19]. In tests of this method, it was possible to distinguish significant differences between the three olibanum species after the dyeing, heating and color development steps. *B. papyrifera* shows a strong blue spot at R_f_ = 0.22 which corresponds to 3-oxo-8, 24-dien-tirucallic acid (elemonic acid) (36), while *B. carterii* and *B. sacra* samples lack this spot. This is consistent with the results of HPLC analysis in which *B. carterii* and *B. sacra* show smaller peak areas for this compound than are found in data from *B. papyrifera* samples. Moreover, when these spot areas are slowly heated after dyeing, the spot corresponding to elemonic acid undergoes a characteristic series of color changes in which it first becomes a greenish spot, and then turns to blue after approximately 24 h before finally turning to brown [19]. This is a useful means of confirming the identity of the spot and further highlights the utility of elemonic acid (36) as a characteristic marker in TLC-based assays to differentiate *B. papyrifera* resins from those obtained from *B. carterii* or *B. sacra*.

Tests of this TLC method led to two other important observations: (i) *B. papyrifera* samples have the strongest AKβ-BA (4) spot, while in *B. serrata* and *B. carterii*/*B. sacra* samples the AKβ-BA (4) spots are weaker and have approximately equal absorbance, and (ii) without the use of UV detection, Kβ-BA (3) is clearly detectable only in *B. papyrifera* and *B. serrata* resin samples, whereas in those from *B. carterii* and *B. sacra* the spot can only be detected by UV at this concentration level [19]. To some extent, these differences may also be helpful in distinguishing *B. papyrifera* resins from those of other species and will encourage future research to establish a more effective method of identifying resins from *Boswellia* species.

### Differentiating “Indian frankincense” from “African frankincense”

A HPLC gradient method [21] has been used to identify the compounds present in “Indian frankincense” and “African frankincense” collected from the *B. serrata* species and the *B. carterii* and *B. frerean* species, respectively. The results showed a significant difference in the ratio of AKβ-BA (4) to Kβ-BA (3). In these resins, AKβ-BA (4) was found to be the predominant compound in African frankincense, while twice as much Kβ-BA (3) was found in Indian frankincense. The ratio of these compounds (4:3) is approximately 0.7 and 4.7 in Indian and African frankincense, respectively [21]. Furthermore, the results indicated that the total amount of pentacyclic triterpenic acids present was approximately 25% lower in Indian frankincense than in African frankincense, but this finding must be regarded with reservation. All in all, “Indian frankincense” (*B. serrata*) and “African frankincense” (*B. carterii* and *B. frereana*) could be approximately differentiated by this HPLC method on the basis of differences in the ratio of AKβ-BA (4) to Kβ-BA (3).

### Pharmacological activities

In view of both the medicinal importance of triterpenoids in the treatment of various chronic diseases and evidence [53,54] suggesting that both natural and synthetic triterpenoids have potential pharmacological activities, our review has so far only scratched the surface of this very fertile field of investigation and has concentrated on the noteworthy triterpenoids. In terms of the pentacyclic triterpenoids, for instance, BAs, lupeolic acids and their derivatives are common compounds with diverse pharmacological activities, including antifungal, antimicrobial, antioxidant [55] and antitumor activities [56]. Similarly, the tetracyclic triterpenoid resinous metabolites also have pharmacological activities. For example, tirucallic acids and their acetoxy- derivatives have been demonstrated to potently inhibit cell proliferation and to induce apoptosis in tumors [9]. Moreover, some compounds (48–53) synthesized from BAs [41-43,48], the characteristic components of frankincense, also show distinct bioactivity and will be introduced in detail (Additional file 1).

**Figure 3:**
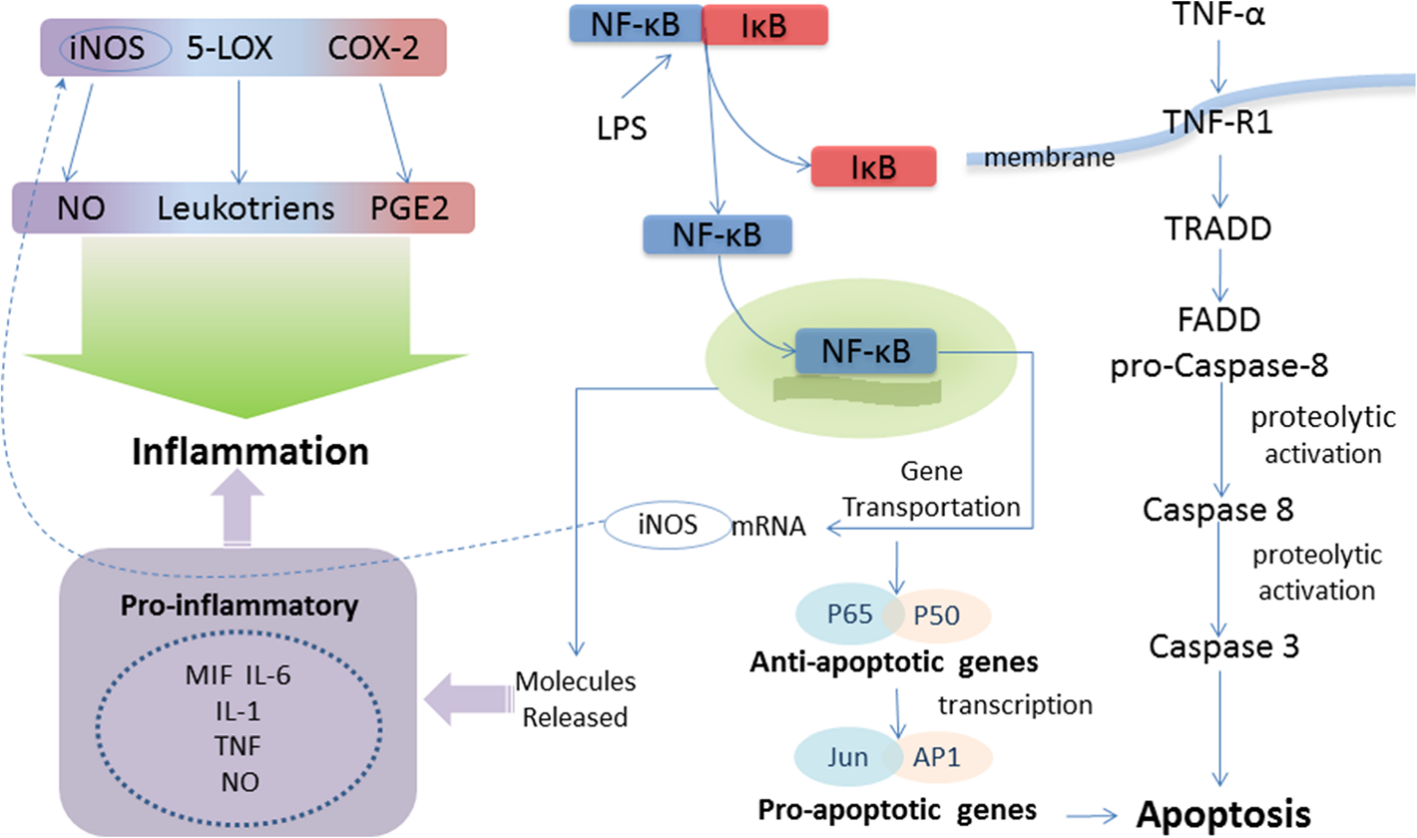
**Some triterpenoids exhibit their anti-inflammatory activity by inhibiting LPS-stimulated COX-2 and iNOS expression in macrophages by limiting the translocation of NF-κB protein into the nucleus.** Signal-induced activation of IκB kinase leads to the phosphorylation and degradation of IκB, liberating NF-κB from the IκB inhibitory proteins. With the degradation of IκB, the NF-κB complex is then freed to enter the nucleus where it can ‘turn on’ the expression of specific genes that have DNA-binding sites for NF-κB nearby and induces the expression of iNOS and the release of proinflammatory such as MIF, IL-6, IL-1, TNF-α. TNF-α is triggering either pro-inflammatory effects via NF-κB related pathways or apoptosis through activation of caspase-8 and triterpenoids induce apoptosis by modulating different caspases and their cleavage. TNF binds its receptor TNF-α and can activate the pro-inflammatory and anti-apoptotic NF-κB pathway via activation of the IKK complex. Moreover, TNF can induce the pro-apoptotic caspase–cascade via adaptor proteins TRADD and FADD and proteolytic activation of procaspase-8. Activated caspase-8 in turn cleaves and thus activates effector caspases such as caspase-3 and induces the apoptosis eventually*.*

On the basis of many studies, the triterpenoid resinous metabolites from the genus *Boswellia* have been shown to exhibit various pharmacological activities *in vitro* and *in vivo* against various health-related conditions, including inflammation, microbial infection and cancer. These pharmacological activities are summarized below, together with the mechanism of action of the triterpenoids (shown in Figure 3).

### Anti-inflammatory activity

Inflammation is a complex process involving numerous mediators with cellular and plasma origins, and these mediators have elaborate, interrelated biological effects [57]. The anti-inflammatory activities of the triterpenoid resinous metabolites are largely ascribed to the inhibition of 5-lipoxygenase (5-LOX), nitric oxide synthase (iNOS), cyclooxygenase-2 (COX-2) and nuclear factor-κB (NF-κB) activities. COX-2 and 5-LOX, two enzymes involved in the oxygenation of arachidonic acid, are upregulated in the central nervous system during aging and are associated with various aging-related brain pathologies [58]. Additionally, excessive and prolonged iNOS-mediated nitric oxide (NO) generation has been linked to both inflammation and tumorigenesis [59]. For clearer understanding of the inflammatory pathogenesis and relevant pro-inflammatory cytokines, its complicated is diagramed in Figure 3 and described in detail below.

Production of eicosanoid compounds, specifically leukotrienes, prostaglandins and lipoxin, is induced by arachidonic acid in the metabolism. Eicosanoids are potent lipid mediators of inflammation that are derived from phospholipase-released arachidonic acid through subsequent metabolism by (COX)-1/2 [60] or LOX and are involved in a variety of homeostatic biological functions and inflammation. With the liberation of arachidonic acid in the cell, several enzymatic reactions take place involving different types of enzymes, such as 12-LOX, 5-LOX, 15-LOX and COX, and each reaction leads to the production of a different type of inflammation mediator. BAs were found to be specific, non-reducing-type inhibitors of the 5-LOX activity that act either by interacting directly with the 5-LOX or by blocking its translocation [31]. Therefore, suppression of leukotriene synthesis via inhibition of 5-LOX is considered to be the main mechanism underlying the anti-inflammatory effect of BAs [61].

COX, also referred to prostaglandins endoperoxide synthase, is a membrane protein which catalyzes the conversion of arachidonic acid and O_2_ to prostaglandins endoperoxide H2, the committed step in prostanoid biosynthesis [62]. Two COX isoforms, COX-1 and COX-2, which are encoded by distinct genes, have been described in mammalian cells. Both isoforms are interesting in the context of both their structural biology and their enzymology in that they are homodimeric, heme-containing, glycosylated proteins with two catalytic sites [62]. These two isozymes are also important pharmacologically as targets of aspirin and other nonsteroidal anti-inflammatory drugs (NSAIDs). COX-2 may be especially important as an NSAID target mediating the inhibition of colon cancer. Although COX-2 is thought to be involved primarily in the inflammatory response, it is also likely to be involved in many essential physiological functions because disruption of the COX-2 gene in mice results in renal dysplasia, cardiac fibrosis, and defects in the ovary [63]. Reports [64,65] have also shown that blockade of COX-1 activity results in multiple severe side effects, such as bleeding in the intestinal tract and impairment of renal function. It is therefore of key importance to note that many studies have demonstrated that certain BAs and their derivatives potently inhibit COX-2 and can also show selective activity against COX-1 [66,67].

Nitric oxide (NO) is an important biological mediator that is synthesized from L-arginine by two isoforms of nitric oxide synthase (NOS), namely constitutive nitric oxide synthase (cNOS) and inducible nitric oxide synthase (iNOS) [57]. At physiological concentrations, NO inhibits proinflammatory platelet aggregation, integrin-mediated adhesion, and proinflammatory-induced gene expression, and these are factors that control vascular inflammation and oxidative injury. However, the overproduction of NO that is catalyzed by iNOS, a soluble enzyme that is active in its dimeric form, is cytotoxic [68]. Immunostimulating cytokines and bacterial pathogens activate iNOS and generate high concentrations of NO through the activation of inducible nuclear factors, including NF-κB [68]. Overall, the overproduction of NO by iNOS is important in inflammation and its related processes, and high levels of NO are markers for the diagnosis of inflammatory disorders. Targeting NF-κB is considered important in inhibiting NOS because it is the main regulatory step in the pathway leading to iNOS expression.

NF-κB (nuclear factor kappa-light-chain-enhancer of activated B cells) is a protein complex that controls the transcription of DNA. Activation of the NF-κB is initiated by the signal-induced degradation of IκB proteins [69]. Signal-induced activation of IκB kinase leads to the phosphorylation and degradation of IκB, liberating NF-κB from the IκB inhibitory proteins [70]. With the degradation of IκB, the NF-κB complex is then freed to enter the nucleus where it can ‘turn on’ the expression of specific genes that have DNA-binding sites for NF-κB nearby [53]. After activation of IκB-NF-κB complex, free NF-κB transfers into the nucleus and induces the expression of iNOS [53,68] and the release of proinflammatory such as MIF, IL-6, IL-1, TNF-α [71] and NO by molecules [53,72] (showed in Figure 3). Again, it is important to note that multiple studies have found that subsets of the resinous triterpenoids are able to suppress NF-κB activation, and we discuss this in more detail below.

### Type U triterpenoid anti-inflammatory activities

The type U triterpenoids β-BA (1) and Aβ-BA (2) inhibit 12-O-tetradecanoylphorbol-13-acetate (TPA)-induced inflammation in mice [50]. Beta-BA (1) can also inhibit the effects of lipopolysaccharide (LPS) through direct molecular interference [73] and has been shown to be a selective inhibitor of COX-1 with IC_50_ values of approximately 15 μM [66]. In addition, β-BA (1) enhances the release of arachidonic acid via cytosolic phospholipase A2 activity, and platelet-type 12-LOX catalysis has been found to increase by approximately 2-fold in its absence, whereas AKβ-BA (4) inhibited platelet-type 12-LOX activity which was identified as a selective molecular target [74]. A form of β-BA (1) lacking β-keto function has been found to mediate only partial inhibition of 5-LOX. Studies [75,76] have revealed that the carboxyl group and the 11-keto group of AKβ-BA (4) in combination are essential for enzyme inhibition, while the acetoxy group at position C-3α increases the affinity of AKβ-BA (4) for the effector site. Moreover, hydrophilic group on C-4 of ring A with the pentacyclic triterpene ring is crucial for binding to the enzyme and for the 5-LOX activity. Furthermore, these reports demonstrated that minor structural modifications could cause a total loss of the binding affinity and/or the inhibitory activity of these compounds. As was reproted that the saponification of AKβ-BA’s 3α-acetoxy-group slightly decreased the activity of (3) (IC_50_ = 3 μM), whereas the substitution with longer aliphatic, lipophilic side chains (e. g. (52), an esterification with butyrylic acid) did not alter the 5-lipoxygenase inhibitory activity [75].

Kβ-BA (3) and AKβ-BA (4) both showed marked anti-inflammatory activity against TPA-induced in mice with a 50% inhibitory dose (ID_50_) of 0.07 and 0.08 (mg/ear) [50]. Additionally, AKβ-BA (4) proved to have significant effects in inhibiting 5-LOX and human leukocyte elastase [8,77] and promoted down-regulating of the expression of COX-2, matrix metalloproteinase-9, C-X-C chemokine receptor 4, and vascular endothelial growth factor *in vivo*[13]. AKβ-BA (4) has also been shown to enhance the release of arachidonic acid via cytosolic phospholipase A2 [74] and has been reported to be a selective inhibitor of COX-1 [66].

12-ursene 2-diketone (5) isolated from *B. serrata* was analyzed for inhibitory effects on key inflammatory mediators, such as TNF-α, IL-1β and IL-6 and the results demonstrated that it can inhibit the expression of pro-inflammatory cytokines and mediators via inhibition of phosphorylation of the mitogen activated protein kinases, *c-jun* N-terminal kinase and p38 [33]. However, no inhibition of extracellular signal-related kinase phosphorylation was observed in LPS-stimulated peripheral blood mononuclear cells [33].

3-acetyl-11α-methoxy-β–BA (6) also shows inhibitory activity against TPA-induced inflammation in mice [50]. α-amyrin (11) and β-amyrin (19) have similar physiological activities in that they affect COX-2 product synthesis slightly [67], while they exhibit pronounced anti-inflammatory effects and suppress the levels of inflammatory cytokines and COX-2 levels. This may occur via inhibition of NF-κB activity and of signaling pathways involving phospho-cyclic AMP response element-binding protein [78]. Coincidently, 3-acetyl-9, 11-dehydro-β-BA (13) and 9, 11-dehydro-β-BA (14) also share similar activity profiles in that both compounds inhibit TPA-induced inflammation in mice [50].

### Type O triterpenoid anti-inflammatory activities

Alpha-BA (16) and Aα-BA (17) show similar inhibitory activity against TPA-induced inflammation in mice [50] and selectively inhibit COX-1 [66]. β-amyrenone (18) and 3-epi-β-amyrin (19) show antifungal and cytotoxic activities in the same range as the organic crude extract and have low-level toxic effects on mononuclear cells obtained from human peripheral blood [79]. Furthermore, olibanumol E (22) inhibits NO production in LPS-activated mouse peritoneal macrophages [80] and also shows inhibitory activity against TPA-induced inflammation in mice [50]. The only artificially synthesized compound belonging to type O was assayed for physiological activity and was found to inhibit the 5-LOX [41].

### Type L triterpenoid anti-inflammatory activities

The type L compounds lupeolic acid (25) and acetyl-lupeolic acid (26), both show inhibitory activity against TPA-induced inflammation in mice [50]. It has been reported that lupenone (27) inhibits protein tyrosine phosphatase 1B [81]. Epi-lupeol (28), which was identified as the principal constituent of *B. frereana*-derived resins, prevents collagen degradation and inhibits the production of pro-inflammatory mediators and matrix metalloproteinase [82]. Lupeol (29) is a multi-target agent with immense anti-inflammatory potential targeting key molecular pathways which involve NF-κB, cFLIP, Fas, Kras, phosphatidylinositol-3-kinase (PI3 κ)/Aκt and Wnt/β-catenin in a variety of cells and its effective therapeutic doses exhibit no toxicity to normal cells and tissues [83]. It has been reported to inhibit NF-κB signaling, including the phosphorylation of the IκB-α protein, and inhibits the binding of the NF-κB complex to DNA, thereby inhibiting NF-κB-dependent reporter gene activity [84-86]. Finally, 3-acetyl-28-hydroxy-lupeolic acid (30) can inhibit the biosynthesis of COX-, 5-LO- and 12-LO-derived eicosanoids from endogenous arachidonic acid in activated platelets, neutrophils, and monocytes from human blood [49].

### Type T triterpenoid anti-inflammatory activities

Alpha-elemolic acid (35), elemonic acid (3-oxo tirucallic acid) (36), 3α-hydroxy-tir-7, 24-dien-21-oic acid (40) and 3α-acetoxy-tirucallic acid (A) (41) show inhibitory activity against 12-O-tetradecanoyl phorbol-13-acetate-induced inflammation in mice [50].

#### Anti-carcinogenic or anti-tumor activities

Resinous triterpenoids, including the BAs, have been suggested to have anti-neoplastic activity as a result of their anti-proliferative and pro-apoptotic properties.

The cytochrome P450 (CYP) superfamily of enzymes plays a central role in the metabolism of carcinogens and anti-cancer drugs [87]. Enhanced expression of CYP in a variety of human cancers suggests that CYP may be a new tumor marker protein, and it has been reported to be an important factor in resistance to anti-cancer drugs [88]. CYP is, therefore, a new and valuable target for anti-cancer strategies.

Epstein–Barr virus, a human herpesvirus, establishes a persistent asymptomatic infection of the circulating B lymphocyte pool [89]. Moreover, it can enhance the carcinogenic effects of viral and chemical carcinogens. The triterpenoids from resins have been studied with regard to their inhibitory effects on TPA-induced Epstein–Barr virus early antigen (EBV-EA) activation and, thus, as potential antitumor agents.

The transcription factor nuclear factor-kappa B (NF-κB) and inhibitor of NF-κB kinase (IKK) proteins are implicated in various cellular processes, including innate- and adaptive-immune responses, cell death and inflammation [69]. However, aberrant and sustained NF-κB or IKK activity has been implicated in various stages of tumorigenesis and is found in a number of cancers [69,90]. NF-κB is activated by Akt and mitogen-activated protein kinase p38, and the expression of these proteins can be decreased to inhibit NF-κB signaling [91].

In addition to interventions in the pathways listed above, topoisomerase IIα, the key enzyme in DNA replication is a molecular target for many anti-cancer drugs called topoII inhibitors [92]. Moreover, modulation of the expression of the let-7 and miR-200 microRNA families [93] and others are also fundamental anti-tumor approaches.

### Type U triterpenoid anti-tumor activities

Beta-BA (1) and Kβ-BA (3) has been reported to possess anti-carcinogenic properties by virtue of its ability to inhibit the activity of applied CYP enzymes with IC_50_ values in the range of 5 μ and 10 μM [94]. It also has moderate inhibitory effects on EBV-EA activation (IC_50_ 431–499 mol ratio/32 pmol TPA). [34]. Moreover, Aβ-BA (2) is cytotoxic for the human glioma cell lines U87 MG and U373 MG [95] and this acetyl-BA derivative interacts with human topoisomerases through high-affinity binding sites yielding KD values of 70.6 nM for topoisomerase I and 7.6 nM for topoisomeraseIIα [95]. In the orthotopic nude mouse model of pancreatic cancer, p.o. administration of AKBA alone (100 mg/kg) significantly inhibited the tumor growth [13]. Moreover, it shows inhibitory effects on EBV-EA induction [34]. AKβ-BA (4), belongs to the type U class of pentacyclic triterpenes and has considerable anti-tumor activity. Firstly, this compound can induce increased expression of the CCAAT/enhancer binding protein homologous protein and DR 5 [8]. Secondly, it inhibits the proliferation of four different pancreatic cancer cell lines (AsPC-1, PANC-28, MIA pancreatic cancer-2 with K-Ras and p53 mutations, and BxPC-3 with wild-type K-Ras and mutated p53) [13]. Thirdly, it induces a decrease in Ki-67 expression, a biomarker of proliferation, and of CD31, a biomarker of microvessel density in tumor tissue [13]. AKβ-BA (4) has also been reported to inhibit human topoisomerases I and IIα [8,95] and may also suppress NF-κB activation [12]. In addition, it proved cytotoxic for the human glioma cell lines U87 MG and U373 MG [95] and all of the three human neuroblastoma cells lines IMR-32, NB-39, and SK-N-SH [34]. Finally, the compound has potent inhibitory effects on applied CYP enzymes with IC_50_ values in the range of 5 μ and 10 μM [94] and exerts antitumor effects on colorectal cancer cells by modulating the expression of the let-7 and miR-200 microRNA families [96]. It has been reported that 3-acetyl-11α-methoxy-β–BA (6) showed similar activity to that of cisplatin against NB-39 and had inhibitory effects on EBV-EA activation [34]. As for the artificially synthesized compound HKBA (51), this can inhibit the enzymatic activity of topoisomerases I and II at 20 μg/ml [42]. In contrast, the BKBA (52) compound functions by inhibiting the NF-κB and STAT proteins, which act both as signal transducers and as activators of transcription, and this compound may therefore be developed into a potential anti-cancer therapeutic agent in the future [43].

### Type O triterpenoid anti-tumor activities

Aside from the aforementioned type U compounds, the triterpenediol (TPD) compounds 3α, 24-dihydroxyurs-12-ene (8) and 3α, 24-dihydroxyolean-12-ene (21), which belong to the type O class, are both able to induce up-regulation of the cell death receptor 4 (DR 4) and TNF-α1, and thereby promote caspase-8 activation and produce oxidative stress in cancer cells [36]. In turn, these changes trigger cell death via activation of both the intrinsic and extrinsic signaling cascades that are regulated by reactive oxygen species and NO. Conversely, these compounds also induce a decrease in the expression of components of the PI3K/pAkt, ERK1/2, and NF-kB/Akt signaling cascades which coordinately contribute to cancer cell survival [10]. The final type O terpenoid we will discuss, AKα-BA (53), exhibits anti-cancer activity and has been shown to inhibit the growth of chemotherapy-resistant human PC-3 prostate cancer cells *in vitro* and to induce apoptosis by activation of caspase 3 and induction of DNA fragmentation [48].

### Type L triterpenoid anti-tumor activities

Lupane triterpenoids are of a particular interest from a medicinal perspective, and their biological activities have attracted attention since the 19th century. For instance, lupeolic acid (25) shows potent inhibitory effects on EBV-EA induction [34]. Lupeol (29) has therapeutic effects in some cancers, in addition to its inhibitory effects on NF-κB signaling described above it seems that it has the potential to inhibit several other signaling pathways, such as the Akt-dependent pathways, and this may enhance its anti-cancer properties [86]. Lupeol (29) has been reported to have a potent inhibitory effect on human leukocytic elastase [97] and was also shown to suppress the growth of HL-60 human leukemia cells by inducing apoptosis [98]. It has also been shown to suppress the malignization of murine melanoma, and it achieves this not only by inhibiting cell proliferation but also by a direct cytotoxic effect [99]. This triterpenoid also has anti-tumor effects on NSGLG-N6 human large cell bronchopulmonary carcinoma [100].

### Type T triterpenoid anti-tumor activities

Elemonic acid (3-oxo-tirucallic acid) (36), β-elemolic acid (37), 3α-hydroxy-tir-7, 24-dien-21-oic acid (40) and 3α-acetoxy-tirucallic acid (A) (41) can show potent inhibitory effects on EBV-EA induction [34]. Furthermore, elemonic acid (3-oxo tirucallic acid) (36) and the acetoxy-derivatives, 3β-acetoxy-tireucallic acid (38) and 3α-acetoxy-tirucallic acid (A) (41) in particular potently inhibited the activities of human recombinant Akt1 and Akt2 and of constitutively active Akt immunoprecipitated from PC-3 cells [9].

### Apoptosis-regulating activities

Apoptosis, or cell suicide, is a form of cell death that is morphologically and biochemically distinct from necrosis [101]. Previous studies have indicated that apoptosis provides a critical regulatory mechanism in inflammatory processes [101] and cancer. Caspase activation plays a central role in the execution of apoptosis and one of the key components of the biochemical pathways of caspase activation is the cell surface death receptor (DR) pathway. In this pathway, activation of caspase-8 following its recruitment to the death-inducing signaling complex is the critical event that transmits the death signal. Activated caspase-8 can activate downstream caspases by direct cleavage or can act indirectly by cleaving Bid and inducing cytochrome *c* activity. Activated caspase-9 then cleaves and activates downstream caspases, such as caspase-3, -6, and −7 [102,103]. Reports [36,104,105] have shown that some of the resinous triterpenoids induce apoptosis in cancer cells and leukemia cell lines by caspase activation. Besides, the pleiotropic cytokine tumor necrosis factor (TNF) alpha is triggering either pro-inflammatory effects via NF-κB related pathways or apoptosis through activation of caspase-8 [103] (the pathway is showed in Figure 3). TNF binds its receptor TNF-α and can activate the pro-inflammatory and anti-apoptotic NF-κB pathway via activation of the IKK complex. Active IKK complexes phosphorylate IκBα resulting in its ubiquitination and thus release and nuclear translocation of NF-κB subunits p50 and p65, where this transcription factor activates pro-inflammatory and anti-apoptotic genes. Moreover, TNF can induce the pro-apoptotic caspase–cascade via adaptor proteins TRADD and FADD and proteolytic activation of procaspase-8. Activated caspase-8 in turn cleaves and thus activates effector caspases such as caspase-3 and induces the apoptosis eventurally [103] (showed in Figure 3).

It is reported that AKβ-BA (4) induces apoptosis through a cells DR 5-mediated pathway in prostate cancer and it activates caspase-8 and caspase-3 in both LNCaP and PC-3 cells [8]. Kβ-BA (3) and AKβ-BA (4) increased caspase-8, caspase-9 and caspase-3 activities accompanied by cleavage of PARP [61]. Moreover, Aα-BA (17) and Akβ-BA (4) convey inhibition of NF-κB and subsequent down-regulation of TNF-α expression in activated human monocytes via their direct inhibitory effects on IKK [106,107].

### Other activities of triterpenoid resinous metabolites

The triterpenoids from the *genus Boswellia* have several physiological activities aside from those already discussed*.* These have been the subject of fewer reports, and we will therefore consider them one-by-one instead of grouping them by type.

Beta-BA (1) exhibited anti-microbial activity against 112 pathogenic bacterial isolates including American Type Culture Collection strains [108]. AKβ-BA(4) was found to be an active compound showing an minimal inhibitory concentration (MIC) range of 2–8 μg/ml against the entire gram positive bacterial pathogens tested. It exhibited concentration-dependent killing of Staphylococcus aureus ATCC 29213 at up to 8 times MIC, and a post-antibiotic effect of 4.8 h at 2 × MIC [108]. It can also act as an antibacterial agent by inhibiting the formation of biofilms generated by S. aureus, S.mutans, Actinomyces and Staphylococcus epidermidis and by reducing preformed biofilms generated by these bacteria [108,109].

Kβ-BA (3), AKβ-BA (4), 3-acetyl-27-hydroxy-lupeolic acid (31), methyl-3α-O-acetyl-27-hydroxy-lupeolic acid (32) and β-Elemolic acid (37) show inhibitory activity against prolyl endopeptidase [7]. Beta-BA(1) can increase the length distribution of microtubule proteins and can also increase the polymerization rate of tubulin, thereby moderately stabilizing it and diminishing both the critical concentration and the fraction of inactive tubulin [110]. Anti-elastase activity has also been reported in the literature [111]. Alpha-amyrenone (9) is a compound that stands out in terms of its activity because of its inhibitory effects on a purified HIV-1 reverse transcriptase [112]. Finally, lupenone (27) inhibits protein tyrosine phosphatase 1B [81], while 3α-acetoxy-tirucallic acid (B) (39) can initiate MEK-1/2 phosphorylation [51]. Moreover a study showed that the mixtures of BAs derive from *B. carterii* have immunomodulatory properties by inhibiting TH1 cytokines and promoting TH2 cytokines *in vitro*[113].

### Safety

In comparison to NSAIDs, which one-third of patients suffer from gastric or duodenal ulcers when taking permanently, the resinous metabolites from *Boswellia* species have attracted increasing popularity in Western countries in the last decade [6]. Several pilot clinical trials suggest promising beneficial therapeutic effects with no serious, long-term, or irreversible adverse effects [114] but some minor adverse effects such as diarrhoea, abdominal pain and nausea [115,116]. A study deals with the evaluation and assessment of the safety/toxic potential of *B. serrata* indicated that *B. serrata* is relatively safe in rat up to the dose of 500 mg/kg B.wt [117]. Another safety profile of alcoholic extract of stem-bark of *B. ovalifoliolata* was investigated and proved the safety with no observed adverse effect level is 500 mg/kg following repeated oral administration for 28 days in rats [118]. In addition, the efficacy, safety and tolerability of *B. serrata* extract was compared with valdecoxib in 66 patients of osteoarthritis of knee for six months, which showed that *B. serrata* extract was superior to valdecoxib in these aspects and only few patients complained of acidity. diarrhea or abdominal cramps [119]. Although the observed adverse effects in further clinical trials with greater numbers of patients is necessary, the previous insights suggested BAs from *Boswellia* to be well tolerated with fewer adverse effects as compared with NSAIDs.

### Bioavailability

On accounts of their potent anti-inflammatory and anti-tumor action as we showed, triterpenoid resinous metabolites from the genus *Boswellia* represent a potential remedy for the complementary treatment of various diseases. However, a major limitation is the low systemic absorption in rodents and humans of BAs, especially of the major active compounds (1–4) (16–17), which have been evidenced by pharmacokinetic studies [120,121]. For example, the plasma and brain concentrations of BAs were found to be very low following oral administration of even high *B. serrata* doses [114]. In general, AKβ-BA (4) was not detected at all [38] while β-BA (1) and Aβ-BA (2) achieved pharmacologically relevant concentrations in plasma [114]. Besides, a permeability study on the intestinal absorption revealed poor permeability (<1 × 10^−6^ cm/s) of AKβ-BA (4) [120] and moderate absorption of Kβ-BA (3) with a P_app_ value of 1.69 × 10^−6^ cm/s in the Caco-2 model [122].

Although the sparing solubility of BAs in water and the highly lipophilic (log *P* = 7-10.3) [123], they can be selectively extracted by using alkyl benzene sulfonate solutions [124]. The hydrotropes cooperatively form microassemblies in aqueous solutions which, in turn, are responsible for the solubilization of water-insoluble organic substances owing to their amphiphilic structures [124]. The solubility of BAs was increased by 2 orders of magnitude in the presence of hydrotropes in aqueous solutions and the efficiency of the extraction depends on the hydrophobic nature of the hydrotrope and also increases with its concentration [124].

To bring the efficacy of triterpenoid resinous metabolites into full play, melioration is necessary by overcoming the limitation of the poor bioavailability. For better prediction of the absorption in *vivo*, the permeability experiments should be more adapted to physiological conditions of the gastrointestinal tract by the addition of 4% bovine serum albumin to the receiver side [120,125], and using modified fasted state simulated intestinal fluid as apical (donor) medium [114]. Under these conditions the four BAs lacking the 11-keto moiety (β-BA (1), Aβ-BA (2), Kβ-BA (3) and AKβ-BA (4)) showed moderate permeability with P_app_-values between 1 and 10 × 10^−6^ cm/s [120], suggesting moderate absorption (20–70%) according to Yee [126]. Furthermore, the permeability of Kβ-BA (3) and AKβ-BA (4) was improved compared to earlier studies from 2.14 × 10^−6^ cm/s to 29.54 × 10^−6^ cm/s for Kβ-BA (3) and from <1 × 10^−6^ cm/s to 17.83 × 10^−6^ cm/s for AKβ-BA (4) [120].

Moreover, a formulation composed of extract/phospholipid/pluronic f127 (1:1:1 w/w/w) increased the solubility of (1–4) (16–17) up to 54 times compared with the nonformulated extract and exhibited the highest mass net flux in the permeability tests [114]. The oral administration of this formulation to rats (240 mg/kg) resulted in 26 and 14 times higher plasma levels for Kβ-BA (3) and AKβ-BA (4), respectively [114]. In the brain, five times higher levels for AKβ-BA (4) compared to the nonformulated extract were determined 8 h after oral administration [114]. Another experiment [120] indicated the availability of all six major BAs (1–4) (16–17) could be confirmed in rat brain 8 h after oral administration of 240 mg/kg gum resin extracts to rats show mean concentrations of 1066.6 ng/g for β-BA (1), 163.7 ng/g for Aβ-BA (2), 11.6 ng/g for Kβ-BA (3), 37.5 ng/g for AKβ-BA (4), 485.1 ng/g for α-BA (16) and 43.0 ng/g for Aα-BA (17). In addition, lecithin formulation significantly improves the absorption of BAs and promotes their tissue penetration [121].

## Review and conclusions

This review summarized a total of 53 compounds and 47 components that have been reported to be among the triterpenoid resinous metabolites from the genus *Boswellia*, and discusses what is known of their physiological activities from more than one hundred published studies. We noted that the triterpenoid resinous metabolites pharmacological activities consisting of anti-inflammatory, anti-carcinogenic, anti-bacterial and apoptosis-regulating activities. Triterpenoid resinous metabolites especially BAs have generated an extensive interest due to the various beneficial pharmacological properties. Besides their potent anti-inflammatory effects, triterpenoids have shown promising activities against multiple malignancies [8-13]. Also, we discussed various mechanisms by which triterpenoids regulate various transcription and growth factors, inflammatory cytokines [127], and intracellular signaling pathways involved in cancer cell apoptosis [53]. They inhibit the production of numerous cytokines [127], inducible enzymes [58,94], and induce apoptosis in tumor cell lines [13,95,104]. Therefore, it has significant potential to serve as a novel agent for cancer prevention and therapy. Among the important chemoprevention and therapeutic target pathways are NF-κB [53], STAT3 [43] and MAP [33]. The extracts of the gum-resin of *Boswellia carteri, Boswellia frereana, Boswellia sacra* and *Boswellia serrata* are identified as equally potent, non-selective inhibitors of the major drug metabolising CYP enzymes [94] and as a selective COX-2 inhibitor [119]. In addition, BAs potently suppressed the proteolytic activity of human cathepsin G (IC_50_ of approximately 600 nM) in a competitive and reversible manner [38].

Several pilot clinical trials suggest promising beneficial therapeutic effects with no serious, long-term, or irreversible adverse effects but some minor adverse effects. This suggested BAs of *Boswellia* to be well tolerated with fewer adverse effects as compared with NSAIDs. Major active compounds (boswellic acids), however, have poor aqueous solubility, which adversely affect its intestinal absorption. This limitation has been addressed by developing a new formulations composed of extract/phospholipid/pluronic f127 (1:1:1 w/w/w) [114].

As we have seen, the triterpenoid resinous metabolites in *Boswellia* have extensive distributions and the phytochemical investigations conducted to date mainly focus on 5 *Boswellia* species, namely *B. carterii, B. serrata, B. sacra, B. papyrifera* and *B. frereana*. The characteristic triterpenoids of these species have potential identifying properties that may be used in efforts to accurately identify the species origins of frankincense. In focusing on the nature and utility of these triterpenoid profiles, our suggested approach offers an alternative to the method of identification used in previous studies, which uses diterpenes and volatiles as the main identifying markers.

On the basis of the experimental evidence, which shows the potential of triterpenoid resinous metabolites as agents for the prevention and treatment of cancer in addition to various diseases associated with inflammation, future studies should be focused on conducting detailed, preclinical studies of triterpenoid toxicity, bioavailability, pharmacodynamics and biomarkers and on extensive evaluation of tumor inhibition using adenocarcinoma as an efficacy end point, before undertaking extensive clinical trials [53]. In addition, existing studies support the assertion that structural modifications of triterpenoids is likely to provide greater bioavailability and efficacy in clinical applications. Above all, more research is needed to clearly identify all of the potential applications of the various species of resinous metabolites that can be isolated from the genus *Boswellia*.

## Abbreviations

5-LOX: 5-lipoxygenase; β-BA: β-Boswellic acid; Aβ-BA: 3-acetyl-β-BA; AKβ-BA: 3-acetyl-11-keto-β-BA; α-BA: α-Boswellic acid; Aα-BA: 3-acetyl α-BA; BAs: Boswellic acids; COX: Cyclooxygenase; CYP: Cytochrome P450; DR: Death receptor; EB-VEA: Epstein–Barr virus early antigen; HKBA: Hexanoyloxy-11-keto-β-BA; HPLC: High performanceliquid chromatography; IC50: Ligand concentration that inhibits enzyme by 50%; IKK: inhibitor of NF-κB kinase; Kβ-BA: 11-keto-β-BA; LPS: Lipopolysaccharide; MIC: Minimal inhibitory concentration; NF-κB: Nuclear factor-κB; NO: Nitric oxide; NSAIDs: Non-steroidal antiinflammatory drugs; PARP: Poly-ADP-ribose polymerase; TLC: Thin-layer chromatography; TNF: Tumor necrosis factor; TPA: 12-O-tetradecanoylphorbol-13-acetate.

## Competing interests

The authors declare that they have no competing interests.

## Authors’ contributions

LY and LC provided the concept and designed the manuscript. ZY has been involved in preparing the manuscript. NZ, ZS, WJ, LB and XX participated in the discussion of views in the paper. All authors have read and approved the final manuscript.

## Supplementary Material

Additional file 1: Table S1 Pharmacological activities of individual compound. Click here for file
