# Supplementary material for: Triterpenoid resinous metabolites from the genus Boswellia: pharmacological activities and potential species-identifying properties
Source: Chem Cent J. 2013 Sep 12;7:153. doi: 10.1186/1752-153X-7-153 (PMC3847453; doi:10.1186/1752-153X-7-153)
Supplement: Additional file 1: Table S1 — Pharmacological activities of individual compound. [file 1752-153X-7-153-S1.docx]

*Supplementary material:*

**Triterpenoid** **resinous metabolites from** **the genus** ***Boswellia*: Pharmacological** **activities** **and potential species-identifying properties**

**Yuxin Zhang^1^, Zhangchi Ning^1^****, Cheng Lu^2^**^^[[1]](#footnote-2)^*^**, Siyu Zhao^1^, Jianfen Wang^1^, Yuanyan Liu^1^**^^[[2]](#footnote-3)^*^

**Affiliation**

^1^ School of Chinese Materia Medica, Beijing University of Chinese Medicine, Beijing, China

^2^ Institute of Basic Research in Clinical Medicine, China Academy of Chinese Medical Sciences, Beijing, China

**Table S1**

| **Constituent** | | **Effect or target** | **Application** |
| --- | --- | --- | --- |
| Tβ-BA**(1)** | | 1. Show inhibitory potential against PEP enzyme. [1] 2. Increase MTP length distribution and the polymerization rate of tubulin, moderately stabilizing it and diminishing both the critical concentration and the fraction of inactive tubulin. [2] 3. Moderate to potent inhibitors of the applied CYP enzymes. [3] 4. Against 112 pathogenic bacterial isolates including ATCC strains. [4] 5. Anti-elastase activity [5] 6. Inhibit lipopolysaccharide functionality through direct molecular interference. [6] 7. COX-1 selective inhibitors. [7] 8. Show moderate inhibitory effects on EBV-EA activation. [8] 9. Enhance the release of arachidonic acid via cytosolic phospholipase A2. [9] 10. Increase platelet-type 12-lipoxygenase catalysis approximately 2-fold in the absence. [9] 11. Show inhibitory activity against 12-*O*-tetradecanoylphorbol-13-acetate(TPA)-induced inflammation in mice. [10] | - 1. A new class of memory enhancing drugs. [11]   2. Having long been used in Ayurveda and Oriental Medicine to prevent amnesia. [2]   3. Having been used as a traditional medicine for the treatment of inflammatory and arthritic diseases. [12]   4. Having anti-carcinogenic, anti-tumor, and anti-hyperlipidemic activities. [12]   5. Exhibit potent cytotoxic activities against all of the three human neuroblastoma cells IMR-32, NB-39, and SK-N-SH. [8] |
| Aβ-BA**(2)** | | 1. Be cytotoxic for the human glioma cell lines U87 MG and U373 MG. [13] 2. COX-1 selective inhibitors. [7] 3. Show potent inhibitory effects on EBV-EA induction. [8] 4. Show inhibitory activity against TPA-induced inflammation in mice. [10] | - 1. Exhibit potent cytotoxic activities against all of the three human neuroblastoma cells IMR-32, NB-39, and SK-N-SH. [8] |
| Kβ-BA**(3)** | | 1. Increase caspase-8, caspase-9 and caspase-3 activities accompanied by cleavage of PARP. [14] 2. Show inhibitory potential against PEP enzyme. [1] 3. Moderate to potent inhibitors of the applied CYP enzymes. [3] 4. Show inhibitory activity against TPA-induced inflammation in mice. [10] | 1. Possessing antiproliferative and apoptotic effects in colon cancer HT-29 cells. [14] 2. Exhibit potent cytotoxic activities against all of the three human neuroblastoma cells IMR-32, NB-39, and SK-N-SH. [8] |
| AKβ-BA**(4)** | | 1. Dual inhibition of 5-LOX and HLE. [15] [16] 2. Activates caspase-8 and caspase-3 as well as PARP cleavage while partially by caspase-9. [16] [14] 3. Increase levels of DR 5. [16] 4. Induce expression of CHOP. [16] 5. Suppresse NF-κB activation[17] 6. Inhibited the proliferation of four different PaCa cell lines (AsPC-1, PANC-28, and MIA PaCa-2 with K-Ras and p53 mutations, and BxPC-3 with wild-type K-Ras and p53 mutation). [18] 7. Decreases in Ki-67, a biomarker of proliferation, and CD31, a biomarker of microvessel density, in the tumor tissue. [17] [18] 8. Downregulate the expression of COX-2, MMP-9, CXCR4, and VEGF in the tissues. [18] 9. COX-1 selective inhibitors. [7] 10. Be cytotoxic for the human glioma cell lines U87 MG and U373 MG. [13] 11. Inhibite human topoisomerases I and IIα. [16] [13] 12. Exhibite concentration dependent killing of Staphylococcus aureus ATCC 29213 up to 8 × MIC and also demonstrated PAE of 4.8 h at 2 × MIC. [4] 13. Inhibite the formation of biofilms generated by S. aureus and Staphylococcus epidermidis and also reduced the preformed biofilms by these bacteria[4] 14. Show prominent inhibitory potential against PEP enzyme. [1] 15. Inhibite the formation of biofilms generated by S.mutans and Actinomyces viscosus and also reduced the preformed biofilms by these bacteria. [19] 16. Moderate to potent inhibitors of the applied CYP enzymes. [3] 17. Exert antitumor effects in colorectal cancer cells by modulating expression of the let-7 and miR-200 microRNA family. [20] 18. Exhibit potent cytotoxic activities against all of the three human neuroblastoma cells IMR-32, NB-39, and SK-N-SH. [8] 19. Enhance the release of arachidonic acid via cytosolic phospholipase A2. [9] 20. Show inhibitory activity against TPA-induced inflammation in mice. [10] | - 1. Induce apoptosis in prostate cancer cells. [16]   2. Affect the growth and metastasis of CRC. [17, 20]   3. Suppresses growth and metastasis of PaCa tumors. [18]   4. Induce apoptosis, and sensitized the cells to apoptotic effects of gemcitabine. [18]   5. Inhibite the metastasis of the PaCa to spleen, liver, and lungs. [18]   6. Possessing antiproliferative and apoptotic effects in colon cancer HT-29 cells. [14]   7. Possessing positive therapeutic effects in IBD. [21]   8. Potential use in treating S. aureus infections. [4]   9. AKβ-BA**(4)** can be further exploited to evolve potential lead compounds in the discovery of new anti-Gram-positive and anti-biofilm agents. [4]   10. Peritumor edema. [19]   11. Exhibite an inhibitory effect on all the oral cavity pathogens tested. [19]   12. Great potential for use in mouthwash for preventing and treating oral infections. [19]   13. Suppress invasion of pancreatic cancer cells through the downregulation of CXCR4 chemokine receptor expression. [22]   14. Having been used in Ayurvedic medicine to treat proinflammatory conditions. [17]   15. AKβ-BA**(4)** is highly effective in suppressing ascites and distant metastasis to the liver, lungs and spleen in orthotopically implanted tumors in nude mice. [17]   16. Treatment of meningioma cells. [23] |
| 12-ursene-2-diketone **(5)** | | - - 1. Inhibit the expression of pro-inflammatory cytokines and mediators via inhibition of phosphorylation of the MAP kinases JNK and p38 while no inhibition was seen in ERK phosphorylation in LPS-stimulated PBMCs. [24] | 1. Block specific cellular targets that are responsible for dopaminergic and cholinergic effects. [25] |
| 3-acetyl-11α-methoxy-β –BA **(6)** | | - - - 1. Exhibite potent cytotoxic activities. [8]       2. Show almost comparable with or higher activity (IC_50_ 13.4-28.2μM) than cisplatin (26.0μM) against NB-39. [8]       3. Show moderate inhibitory effects on EBV-EA activation. [8]       4. Show inhibitory activity against TPA-induced inflammation in mice. [10] | 1. Exhibit potent cytotoxic activities against all of the three human neuroblastoma cells IMR-32, NB-39, and SK-N-SH. [8] |
| TPD | 3α,24-dihydroxyurs-12-ene **(8)** | 1. TPD up regulated the expression of cell death receptors DR4 and TNF-R1 level, leading to caspase-8 activation. [26] 2. TPD produces oxidative stress in cancer cells that triggers self-demise by ROS and NO regulated activation of both the intrinsic and extrinsic signaling cascades. [26] 3. Decrease the expression of PI3K/pAkt, ERK1/2, NF-kB/Akt signaling cascades which coordinately contribute to cancer cell survival through these distinct pathways. [27] | 1. Induce apoptosis through both the intrinsic and extrinsic apoptotic pathways in human leukemia HL-60 cells. [26] 2. Apoptotic cell death in human cervical cancer HeLa and SiHa cells. [27] 3. The tumor suppressor p53 pathway predominantly activated by TPD further up-regulated PUMA, which concomitantly decreased the Bcl-2 level, caused mitochondrial membrane potential loss with attendant translocation of Bax and drp1 to mitochondria and release of pro-apoptotic factors such as cytochrome c and Smac/Diablo to cytosol leading to caspases-3 and - 9 activation. [27] |
|  | 3α,24-dihydroxyolean-12-ene**(20)** |  |  |
| α-amyrenone **(9)** | | 1. Exhibit inhibitory effects on a purified HIV-1 reverse transcriptase. [28] | ___________ |
| α-amyrin **(11)** | | 1. Affected COX-2 product synthesis slightly. [29] 2. Exhibit pronounced anti-inflammatory effects. [30] 3. Suppression of inflammatory cytokines and COX-2 levels, possibly via inhibition of NF-κB and CREB-signalling pathways. [30] | 1. A potential use to control inflammatory responses in bowel disease. [30] 2. Systemic administration exerted a marked and rapid inhibition of TNBS-induced colitis. [30] 3. Antinociceptive properties. [31] 4. A natural triterpenoid ameliorates L-arginine induced acute pancreatitis in rats. [32] |
| β-amyrin **(19)** | |  |  |
| 3-acetyl-9,11-dehydro-β-BA **(13)** | | 1. Show inhibitory activity against TPA-induced inflammation in mice. [10] | 1. Exhibit potent cytotoxic activities against all of the three human neuroblastoma cells IMR-32, NB-39, and SK-N-SH. [8] |
| 9,11-dehydro-β-BA **(14)** | |  |  |
| α-BA **(15)** | | 1. Show inhibitory activity against TPA-induced inflammation in mice. [10] |  |
| Aα-BA **(16)** | | 1. Inhibite human topoisomerases I and IIα. [13] 2. COX-1 selective inhibitors. [7] 3. Exhibit potent cytotoxic activities against all of the three human neuroblastoma cells IMR-32, NB-39, and SK-N-SH. [8] 4. Show inhibitory activity against TPA-induced inflammation in mice. [10] | - 1. Might be used as anti-cancer agents. [33] |
| β-amyrenone **(17)** | | 1. Show antifungal and cytotoxic activities in the same range as the organic crude extract and low toxic effect against mononuclear cells obtained from human peripheral blood. [34] | ___________ |
| 3-epi-β-amyrin **(18)** | |  |  |
| olibanumol E **(21)** | | 1. Exhibite nitric oxide production inhibitory activity in lipopolysaccharide-activated mouse peritoneal macrophages. [35] | 1. Anti-inflammation. |
| lupeolic acid **(25)** | | 1. Show potent inhibitory effects on EBV-EA induction. [8] 2. Show inhibitory activity against TPA-induced inflammation in mice. [10] | 1. Exhibit potent cytotoxic activities against all of the three human neuroblastoma cells IMR-32, NB-39, and SK-N-SH. [8] |
| acetyl-lupeolic acid **(26)** | |  |  |
| lupenone **(27)** | | - - - 1. Inhibition of protein tyrosine phosphatase 1B. [36] | 1. Anti-Inflammatory and Antiulcer Activities. [37] |
| epi- lupeol **(28)** | | 1. Identified the principal constituent of B. frereana which prevents collagen degradation, and inhibits the production of pro-inflammatory mediators and MMPs. [38] | 1. A potential therapeutic agent for treating inflammatory symptoms associated with arthritis. [38] |
| Lupeol **(29)** | | 1. Inhibit NF-kB signaling, including phosphorylation of IkBa protein, DNA binding of NF-kB complex and NF-kB-dependent reporter gene activity. [39-41] 2. Suppress the growth of HL-60 human leukemia cells by inducing their apoptosis. [42] | 1. A high activity against NSGLG-N6 human large cell bronchopulmonary carcinoma. [43] 2. Prevent cancer, coronary and hepatic diseases[44] |
| 3-acetyl-28-hydroxy-lupeolic acid **(30)** | | 1. Inhibit the biosynthesis of COX-, 5-LO- and 12-LO-derived eicosanoids from endogenous arachidonic acid in activated platelets, neutrophils, and monocytes from human blood. [45] |  |
| 3-acetyl-27-hydroxy-lupeolic acid **(31)** | | 1. Show more active inhibitory potential against PEP enzyme even than AKβ-BA **(4)**. [1] | 1. A new class of memory enhancing drugs. [11] |
| methyl-3α-O-acetyl-27-hydroxy- lupeolic acid **(32)** | | 1. Show inhibitory potential against PEP enzyme. [1] | 1. A new class of memory enhancing drugs. [11] |
| olibanumol F **(33)** olibanumol G **(34)** | | No remarkable result [35] | |
| α-Elemolic acid **(35)** | | 1. Show inhibitory activity against 12-O-tetradecanoyl phorbol-13-acetate-induced inflammation in mice. [10] | _________ |
| Elemonic acid (3-oxo tirucallic acid) **(36)** | | 1. Inhibited the activities of human recombinant Akt1 and Akt2. [46] 2. Show potent inhibitory effects on EBV-EA induction. [8] 3. Show inhibitory activity against TPA-induced inflammation in mice. [10] | 1. A new class of Akt inhibitors with antitumor properties. [46] 2. Exhibit potent cytotoxic activities against all of the three human neuroblastoma cells IMR-32, NB-39, and SK-N-SH. [8] |
| β- Elemolic acid **(37)** | | 1. Show inhibitory potential against PEP enzyme. [1] 2. Show potent inhibitory effects on EBV-EA induction. [8] | 1. A new class of memory enhancing drugs. [11] |
| 3β-acetoxy-tireucallic acid **(38)** | | 1. Inhibited the activities of human recombinant Akt1 and Akt2. [46] | 1. A new class of Akt inhibitors with antitumor properties. [46] |
| 3α-acetoxy-tirucallic acid(B) **(39)** | | 1. Initiate MEK-1/2 phosphorylation. [47] | _________ |
| 3α-hydroxy-tir-7,24-dien-21-oic acid **(40)** | | 1. Show potent inhibitory effects on EBV-EA induction. [8] 2. Show inhibitory activity against TPA-induced inflammation in mice. [10] | 1. Exhibit potent cytotoxic activities against all of the three human neuroblastoma cells IMR-32, NB-39, and SK-N-SH. [8] |
| 3α-acetoxy-tirucallic acid(A) **(41)** | | 1. Inhibited the activities of human recombinant Akt1 and Akt2. [46] 2. Show potent inhibitory effects on EBV-EA induction. [8] 3. Show inhibitory activity against TPA-induced inflammation in mice. [10] | 1. A new class of Akt inhibitors with antitumor properties. [46] 2. Exhibit potent cytotoxic activities against all of the three human neuroblastoma cells IMR-32, NB-39, and SK-N-SH. [8] |
| * 11-keto-diol **(48)** | | 1. Inhibit the 5-LOX activity. [48] |  |
| * 11-keto-β-BA methyl ester **(49)** | | No remarkable result [35] | |
| * acetyl-11-keto-amyrin **(50)** | |  |  |
| * HKBA **(51)** | | 1. Inhibite the enzymatic activity of topoisomerases I and II. [49] | 1. Might be used as anti-cancer agents. [33] |
| * BKBA **(52)** | | 1. Exhibit anti-cancer activity by inhibiting the NF-κB and STAT proteins. [50] | 1. Develope into a potential anti-cancer therapeutic. [50] |
| *AKα-BA **(53)** | | 1. Inhibit the growth of chemotherapy-resistant human PC-3 prostate cancer cells *in vitro* and induces apoptosis as shown by activation of caspase 3 and the induction of DNA fragmentation. [51] | 1. Be active *in vivo* as shown by inhibition of proliferation and induction of apoptosis in PC-3 prostate cancer cells xenotransplanted onto the chick chorioallantoic membrane. [51] |
| 2α,3α-dihydroxy-urs-12-en-24-oic acid **(7)** 3-epi-α-amyrin **(10)** 3-acetyl-11-hydroxy-BA **(12)** 9,11-dehydro-α-BA **(22)** 3-acetyl-9,11-dehydro-α-BA **(23)** 18Hα,3β,20β-ursanediol **(24)** Not tested | | | |

**Abbreviations:**

| 5-LOX | 5-lipoxygenase |
| --- | --- |
| β-BA | β-Boswellic acid |
| Aβ-BA | 3-acetyl-β-BA |
| AKβ-BA | 3-acetyl-11-keto-β-BA |
| α-BA | α-Boswellic acid |
| Aα-BA | 3-acetyl α-BA |
| AD | Alzheimer‘s disease |
| ATCC | American Type Culture Collection |
| BAs | Boswellic acids |
| BKBA | Butyryloxy-11-keto-β-BA |
| CHOP | CCAAT/enhancer binding protein homologous protein |
| COX | Cyclooxygenase |
| CRC | Colorectal cancer |
| CREB | Phospho-cyclic amp response element-binding protein |
| CXCR | C-X-C chemokine receptor |
| CYP | Cytochrome P450 |
| DR | Death receptor |
| EB-VEA | Epstein–Barr virus early antigen |
| ERK | Extracellular signal related kinase |
| HIV1 | Human immunodeficiency virus type 1 |
| HKBA | Hexanoyloxy-11-keto-β-BA |
| HLE | Human leukocyte elastase |
| IBD | Inflammatory bowel disease |
| IC_50_ | Ligand concentration that inhibits enzyme by 50% |
| Kβ-BA | 11-keto-β-BA |
| LNCaP | Lymph node carcinoma of prostate |
| LPS | Lipopolysaccharide |
| MAP | Mitogen activated protein |
| MIC | Minimal inhibitory concentration |
| MMP | Matrix metalloproteinas |
| MTP | Microtubule protein |
| NF-κB | Nuclear factor-κB |
| PaCa | Pancreatic cancer |
| PAE | Postantibiotic effect |
| PARP | Poly-ADP-ribose polymerase |
| PBMCs | Peripheral blood mononuclear cells |
| PC | Proprotein convertases |
| PEP | Prolyl endopeptidase |
| RP-HPLC | Reversed-phase high-performance liquid chromatograph |
| TLC | Thin-layer chromatography |
| TNBS | Trinitrobenzene sulphonic acid |
| TPD | Triterpenediol |
| VEGF | Vascular endothelial growth factor |

**References:**

1.Atta-ur-Rahman, Naz H, Fadimatou, Makhmoor T, Yasin A, Fatima N, Ngounou F, Kimbu S, Sondengam B and Choudhary MI: **Bioactive Constituents from *Boswellia* papyrifera.** *Journal of natural products* 2005, **68**: 189-193.

2.Karima O, Riazi G, Khodadadi S, Yousefi R, Mahnam K, Mokhtari F, Cheraghi T and Moosavi-Movahedi AA: **An *in vitro* study of the role of β-boswellic acid in the microtubule assembly dynamics.** *FEBS letters* 2012, **586:** 4132–4138.

3.Frank A and Unger M: **Analysis of frankincense from various *Boswellia* species with inhibitory activity on human drug metabolising cytochrome P450 enzymes using liquid chromatography mass spectrometry after automated on-line extraction.** *Journal of Chromatography A* 2006, **1112**: 255-262.

4.Raja AF, Ali F, Khan IA, Shawl AS, Arora DS, Shah BA and Taneja SC: **Antistaphylococcal and biofilm inhibitory activities of acetyl-11-keto-β-boswellic acid from *Boswellia serrata*.** *BMC microbiology* 2011, **11**: 54.

5.Thring TS, Hili P and Naughton DP: **Anti-collagenase, anti-elastase and anti-oxidant activities of extracts from 21 plants.** *BMC Complementary and Alternative Medicine* 2009, **9**: 27.

6.Henkel A, Kather N, Mönch B, Northoff H, Jauch J and Werz O: **Boswellic acids from frankincense inhibit lipopolysaccharide functionality through direct molecular interference.** *Biochemical Pharmacology* 2012, **83**:115-121.

7.Cao H, Yu R, Choi Y, Ma ZZ, Zhang H, Xiang W, Lee DYW, Berman BM, Moudgil KD and Fong HHS: **Discovery of cyclooxygenase inhibitors from medicinal plants used to treat inflammation.** *Pharmacological Research* 2010, **61**: 519-524.

8.Akihisa T, Tabata K, Banno N, Tokuda H, Nishihara R, Nakamura Y, Kimura Y, Yasukawa K and Suzuki T: **Cancer chemopreventive effects and cytotoxic activities of the triterpene acids from the resin of *Boswellia* *carteri*.** *Biological and Pharmaceutical Bulletin* 2006, **29**: 1976-1979.

9.Poeckel D, Tausch L, Kather N, Jauch J and Werz O: **Boswellic acids stimulate arachidonic acid release and 12-lipoxygenase activity in human platelets independent of Ca^2+^ and differentially interact with platelet-type 12-lipoxygenase.** *Molecular pharmacology* 2006, **70**: 1071-1078.

10.Banno N, Akihisa T, Yasukawa K, Tokuda H, Tabata K, Nakamura Y, Nishimura R, Kimura Y and Suzuki T: **Anti-inflammatory activities of the triterpene acids from the resin of *Boswellia* *carteri*.** *Journal of ethnopharmacology* 2006, **107**: 249.

11.De Nanteuil G, Portevin B and Lepagnol J: **Prolyl endopeptidase inhibitors: a new class of memory enhancing drugs.** *Drugs of the Future* 1998, **23**: 167-180.

12.Huang MT, Badmaev V, Ding Y, Liu Y, Xie JG and Ho CT: **Anti-tumor and anti-carcinogenic activities of triterpenoid, β-boswellic acid.** *BioFactors* 2000, **13**: 225-230.

13.Syrovets T, Büchele B, Gedig E, Slupsky JR and Simmet T: **Acetyl-boswellic acids are novel catalytic inhibitors of human topoisomerases I and IIα.** *Molecular pharmacology* 2000, **58**: 71-81.

14.Liu JJ, Nilsson Å, Oredsson S, Badmaev V, Zhao WZ and Duan RD: **Boswellic acids trigger apoptosis via a pathway dependent on caspase-8 activation but independent on Fas/Fas ligand interaction in colon cancer HT-29 cells.** *Carcinogenesis* 2002, **23**: 2087-2093.

15.Safayhi H, Rall B, Sailer E-R and Ammon HPT: **Inhibition by boswellic acids of human leukocyte elastase.** *Journal of Pharmacology and Experimental Therapeutics* 1997, **281**: 460-463.

16.Lu M, Xia L, Hua H and Jing Y: **Acetyl-Keto-β-Boswellic Acid Induces Apoptosis through a Death Receptor 5–Mediated Pathway in Prostate Cancer Cells.** *Cancer research* 2008, **68**: 1180-1186.

17.Yadav VR, Prasad S, Sung B, Gelovani JG, Guha S, Krishnan S and Aggarwal BB: **Boswellic acid inhibits growth and metastasis of human colorectal cancer in orthotopic mouse model by downregulating inflammatory, proliferative, invasive and angiogenic biomarkers.** *International Journal of Cancer* 2011, **130**: 2176-2184.

18.Park B, Prasad S, Yadav V, Sung B and Aggarwal BB: **Boswellic acid suppresses growth and metastasis of human pancreatic tumors in an orthotopic nude mouse model through modulation of multiple targets.** *PloS one* 2011, **6**: e26943.

19.Raja AF, Ali F, Khan IA, Shawl AS and Arora DS: **Acetyl-11-keto-β-boswellic acid (AKBA); targeting oral cavity pathogens.** *BMC research notes* 2011, **4**: 406.

20.Takahashi M, Sung B, Shen Y, Hur K, Link A, Boland CR, Aggarwal BB and Goel A: **Boswellic acid exerts anti-tumor effects in colorectal cancer cells by modulating expression of the let-7 and miR-200 microRNA family.** *Carcinogenesis* 2012, **33**: 2441-2449.

21.Krieglstein CF, Anthoni C, Rijcken EJM, Laukötter M, Spiegel HU, Boden SE, Schweizer S, Safayhi H, Senninger N and Schürmann G: **Acetyl-11-keto-ß-boswellic acid, a constituent of a herbal medicine from *Boswellia serrata* resin, attenuates experimental ileitis.** *International journal of colorectal disease* 2001, **16**: 88-95.

22.Park B, Sung B, Yadav VR, Cho SG, Liu M and Aggarwal BB: **Acetyl-11-keto-β-boswellic acid suppresses invasion of pancreatic cancer cells through the downregulation of CXCR4 chemokine receptor expression.** *International Journal of Cancer* 2011, **129**: 23-33.

23.Park YS, Lee JH, Bondar J, Harwalkar JA, Safayhi H and Golubic M: **Cytotoxic action of acetyl-11-keto-β-boswellic acid (AKBA) on meningioma cells.** *Planta medica* 2002, **68**: 397-401.

24.Gayathri B, Manjula N, Vinaykumar K, Lakshmi B and Balakrishnan A: **Pure compound from *Boswellia serrata* extract exhibits anti-inflammatory property in human PBMCs and mouse macrophages through inhibition of TNFα, IL-1β, NO and MAP kinases.** *International immunopharmacology* 2007, **7**: 473-482.

25.Shah SA, Rathod IS, Suhagia BN, Patel DA, Parmar VK, Shah BK and Vaishnavi VM: **Estimation of boswellic acids from market formulations of *Boswellia serrata* extract and 11-keto β-boswellic acid in human plasma by high-performance thin-layer chromatography.** *Journal of Chromatography B* 2007, **848**: 232-238.

26.Bhushan S, Kumar A, Malik F, Andotra SS, Sethi VK, Kaur IP, Taneja SC, Qazi GN and Singh J: **A triterpenediol from *Boswellia serrata* induces apoptosis through both the intrinsic and extrinsic apoptotic pathways in human leukemia HL-60 cells.** *Apoptosis* 2007, **12**: 1911-1926.

27.Bhushan S, Malik F, Kumar A, Isher HK, Kaur IP, Taneja SC and Singh J: **Activation of p53/p21/PUMA alliance and disruption of PI-3/Akt in multimodal targeting of apoptotic signaling cascades in cervical cancer cells by a pentacyclic triterpenediol from *Boswellia serrata*.** *Molecular carcinogenesis* 2009, **48**: 1093-1108.

28.Akihisa T, Ogihara J, Kato J, Yasukawa K, Ukiya M, Yamanouchi S and Oishi K: **Inhibitory effects of triterpenoids and sterols on human immunodeficiency virus-1 reverse transcriptase.** *Lipids* 2001, **36**: 507-512.

29.Siemoneit U, Hofmann B, Kather N, Lamkemeyer T, Madlung J, Franke L, Schneider G, Jauch J, Poeckel D and Werz O: **Identification and functional analysis of cyclooxygenase-1 as a molecular target of boswellic acids.** *Biochemical Pharmacology* 2008, **75**: 503-513.

30.Vitor C, Figueiredo C, Hara D, Bento A, Mazzuco T and Calixto J: **Therapeutic action and underlying mechanisms of a combination of two pentacyclic triterpenes, α-and β-amyrin, in a mouse model of colitis.** *British journal of pharmacology* 2009, **157**: 1034-1044.

31.Otuki MF, Ferreira J, Lima FV, Meyre-Silva C, Malheiros Â, Muller LA, Cani GS, Santos AR, Yunes RA and Calixto JB: **Antinociceptive properties of mixture of α-amyrin and β-amyrin triterpenes: evidence for participation of protein kinase C and protein kinase A pathways.** *Journal of Pharmacology and Experimental Therapeutics* 2005, **313**: 310-318.

32.Melo CM, Carvalho KMMB, de Sousa Neves JC, Morais TC, Rao VS, Santos FA, de Castro Brito GA and Chaves MH: **α, β-amyrin, a natural triterpenoid ameliorates L-arginine-induced acute pancreatitis in rats.** *World journal of gastroenterology: WJG* 2010, **16**: 4272.

33.Wasserman RA, Austin CA, Fisher LM and Wang JC: **Use of yeast in the study of anticancer drugs targeting DNA topoisomerases: expression of a functional recombinant human DNA topoisomerase IIα in yeast.** *Cancer research* 1993, **53**: 3591-3596.

34.Cota BB, Johann S, Oliveira DM, Siqueira EP, Souza-Fagundes EM, Cisalpino PS, Alves T and Zani CL: **Biological potential of Stillingia oppositifolia.** *Revista Brasileira de Farmacognosia* 2011, **21**: 70-77.

35.Yoshikawa M, Morikawa T, Oominami H and Matsuda H: **Absolute stereostructures of olibanumols A, B, C, H, I, and J from olibanum, gum-resin of *Boswellia* *carterii*, and inhibitors of nitric oxide production in lipopolysaccharide-activated mouse peritoneal macrophages.** *Chemical and Pharmaceutical Bulletin* 2009, **57**: 957-964.

36.Na M, Kim BY, Osada H and Ahn JS: **Inhibition of protein tyrosine phosphatase 1B by lupeol and lupenone isolated from Sorbus commixta.** *Journal of enzyme inhibition and medicinal chemistry* 2009, **24**: 1056-1059.

37.Flekhter O, Nigmatullina L, Karachurina L, Baltina L, Zarudii F, Davydova V, Galin F and Tolstikov G: **The Synthesis and the Anti-Inflammatory and Antiulcer Activities of a Number of 2-Substituted Derivatives of Betulonic Acid, Methylbetulone, and Lupenone.** *Pharmaceutical Chemistry Journal* 2000, **34**: 588-591.

38.Blain EJ, Ali AY and Duance VC: ***Boswellia* *frereana* (frankincense) suppresses cytokine-induced matrix metalloproteinase expression and production of pro-inflammatory molecules in articular cartilage.** *Phytotherapy Research* 2009, **24**: 905-912.

39.Sun B and Karin M: **NF-κB signaling, liver disease and hepatoprotective agents.** *Oncogene* 2008, **27**: 6228-6244.

40.Belakavadi M and Salimath BP: **Mechanism of inhibition of ascites tumor growth in mice by curcumin is mediated by NF-kB and caspase activated DNase.** *Molecular and cellular biochemistry* 2005, **273**: 57-67.

41.Salminen A, Lehtonen M, Suuronen T, Kaarniranta K and Huuskonen J: **Terpenoids: natural inhibitors of NF-κB signaling with anti-inflammatory and anticancer potential.** *Cellular and molecular life sciences* 2008, **65**: 2979-2999.

42.Saleem M: **Lupeol, a novel anti-inflammatory and anti-cancer dietary triterpene.** *Cancer letters* 2009, **285**: 109-115.

43.Tolstikova T, Sorokina I, Tolstikov G, Tolstikov A and Flekhter O: **Biological activity and pharmacological prospects of lupane terpenoids: I. Natural lupane derivatives.** *Russian Journal of Bioorganic Chemistry* 2006, **32**: 37-49.

44.Wal P, Wal A, Sharma G and Rai A: **Biological activities of lupeol.** *Systematic Reviews in Pharmacy* 2011, **2**: 96.

45.Verhoff M, Seitz S, Northoff H, Jauch J, Schaible AM and Werz O: **A novel C (28)-hydroxylated lupeolic acid suppresses the biosynthesis of eicosanoids through inhibition of cytosolic phospholipase A_2_.** *Biochemical Pharmacology* 2012, **84**: 681-691.

46.Estrada AC, Syrovets T, Pitterle K, Lunov O, Büchele B, Schimana-Pfeifer J, Schmidt T, Morad SAF and Simmet T: **Tirucallic acids are novel pleckstrin homology domain-dependent Akt inhibitors inducing apoptosis in prostate cancer cells.** *Molecular pharmacology* 2010, **77**: 378-387.

47.Boden SE, Schweizer S, Bertsche T, Düfer M, Drews G and Safayhi H: **Stimulation of leukotriene synthesis in intact polymorphonuclear cells by the 5-lipoxygenase inhibitor 3-oxo-tirucallic acid.** *Molecular pharmacology* 2001, **60**: 267-273.

48.Sailer ER, Subramanian LR, Rall B, Hoernlein RF, Ammon H and Safayhi H: **Acetyl-11-keto-β-boswellic acid (AKBA): structure requirements for binding and 5-lipoxygenase inhibitory activity.** *British journal of pharmacology* 2012, **117**: 615-618.

49.Chashoo G, Singh SK, Mondhe DM, Sharma PR, Andotra SS, Shah BA, Taneja SC and Saxena AK: **Potentiation of the antitumor effect of 11-keto-β-boswellic acid by its 3-α-hexanoyloxy derivative.** *European journal of pharmacology* 2011, **668**: 390-400.

50.Kumar A, Shah BA, Singh S, Hamid A, Singh SK, Sethi VK, Saxena AK, Singh J and Taneja SC: **Acyl derivatives of boswellic acids as inhibitors of NF-κB and STATs.** *Bioorganic & medicinal chemistry letters* 2012, **22**:431-435.

51.Buchele B, Zugmaier W, Estrada A, Genze F, Syrovets T, Paetz C, Schneider B and Simmet T: **Characterization of 3alpha-Acetyl-11-keto-alpha-boswellic Acid, a Pentacyclic Triterpenoid Inducing Apoptosis *in vitro* and *in vivo*.** *Planta medica* 2006, **72**: 1285.

1. [↑](#footnote-ref-2)
2. * *Corresponding author*:

   Dr. Yuanyan Liu, School of Chinese Materia Medica, Beijing University of Chinese Medicine, Beijing 100029, China. Tel: +86 10 84738658, Fax: +86 10 84738611.

   *E-mail address*: [yyliu_1980@163.com](mailto:yyliu_1980@163.com) (Y.Y. Liu)

   Dr. Cheng Lu, Institute of Basic Research in Clinical Medicine, China Academy of Chinese Medical Sciences, Beijing 100700, China. Tel.: +86 10 64014411-3403, Fax: +86 10 84032881.

   *E-mail address*: [lv_cheng0816@163.com](mailto:lv_cheng0816@163.com) (C. Lu). [↑](#footnote-ref-3)
